# Peripheral Blood Endothelial Progenitors as Potential Reservoirs of Kaposi's Sarcoma-Associated Herpesvirus

**DOI:** 10.1371/journal.pone.0001520

**Published:** 2008-01-30

**Authors:** Silvia Della Bella, Adriano Taddeo, Maria L. Calabrò, Lucia Brambilla, Monica Bellinvia, Elisa Bergamo, Mario Clerici, Maria L. Villa

**Affiliations:** 1 Laboratory of Immunology, Dipartimento di Scienze e Tecnologie Biomediche, Università degli Studi di Milano, Milan, Italy; 2 UOC Immunology and Diagnostic Molecular Oncology, Istituto Oncologico Veneto, IRCCS, Padova, Italy; 3 Department of Dermatology, IRCCS Ospedale Maggiore, Milan, Italy; 4 Centro Santa Maria Nascente, IRCCS Fondazione Don Gnocchi, Milan, Italy; Institut Pasteur Korea, Republic of Korea

## Abstract

**Background:**

The cellular reservoirs of Kaposi's sarcoma-associated herpesvirus (KSHV) and the exact nature of the putative KSHV-infected circulating precursor of spindle cells of Kaposi's sarcoma (KS) still remain poorly defined. Because KS spindle cells are thought to be of endothelial origin, and because mature endothelial cells do not sustain persistent KSHV-infection, our attention was focalized on circulating hematopoietic precursors able to differentiate into endothelial lineage.

**Methods and Findings:**

Late endothelial progenitor cells (late-EPCs) were cultured from the peripheral blood mononuclear cells of 16 patients with classic KS. The presence and load of KSHV genomes were analyzed by real-time polymerase chain reaction in DNA extracted from cells and supernatants of late-EPC cultures obtained from 7 patients. Endothelial colonies cultured from the peripheral blood of KS patients were found to satisfy all requisites to be defined late-EPCs: they appeared from the CD14-negative fraction of adherent cells after 11–26 days of culture, could be serially expanded in vitro, expressed high levels of endothelial antigens but lacked leukocyte markers. Late-EPC cultures were found to harbor KSHV-DNA at variable levels and to retain the virus after multiple passages in cells as well as in supernatants, suggesting that a quote of KSHV lytic infection may spontaneously occur. Lytic phase induction or hypoxia could amplify virus release in supernatants.

**Conclusion:**

Our results suggest that circulating endothelial progenitors from KS patients are KSHV-infected and support viral productive replication and may therefore represent potential virus reservoirs and putative precursors of KS spindle cells.

## Introduction

Kaposi's sarcoma (KS) is a multifocal angioproliferative disease of the skin and mucosa. Central to its pathogenesis is a hyperproliferation of spindle-shaped cells, which are the prominent and hallmark cells of KS lesions. Spindle cells are thought to be of endothelial origin that assume the characteristic spindle shape upon infection with KS-associated herpesvirus (KSHV), also called human herpesvirus-8 (HHV-8), the causative agent for KS [1]. The cellular reservoirs of KSHV infection still remain poorly defined. Previous investigations have demonstrated KSHV infection in circulating monocyte/macrophages and B lymphocytes of KS patients, and suggested therefore that these cells may participate in the dissemination of viral infection in vivo [2]. More recent studies have demonstrated that also progenitor populations, as CD34+ cells, can sustain persistent KSHV infection when infected in vitro, and may therefore act as possible infection reservoir [3].

In KS patients multiple lesions, supposed to be multiclonal in origin [4], can appear synchronously in widely dispersed areas, without evidence of a primary tumor as a source of metastasis. Although it cannot be excluded that KSHV may directly infect mature endothelial cells at the site of the lesions, two main considerations rather suggest that KS lesions may originate from the seeding of previously infected endothelial precursors. The first consideration derives from the observation that KS lesions often progress during or following states of systemic inflammation, and that KS tumors sometimes arise precisely at sites of previous local inflammation, such as surgical wounds (a property known as the Koebner phenomenon) [5], thus suggesting that an inflammatory environment can elicit spindle cell proliferation from circulating KSHV-infected precursors. The second and more stringent consideration derives from the demonstration that recipients of kidney allografts KSHV-negative prior to transplantation may develop KS lesions containing KSHV-infected neoplastic cells of donor origin. This observation clearly indicates that infected progenitor cells from KSHV-infected donors without KS can be seeded and undergo neoplastic transformation and progression in the recipient immunosuppressed hosts [6]. Although it is possible that donor-derived KS precursors might be mature endothelial cells of the kidney, it appears far more likely that they may originate from circulating endothelial progenitors entrapped within the graft.

A clear-cut identification of putative KSHV-infected endothelial progenitors in the peripheral blood of KS patients is still lacking. Therefore, in the present study we cultured circulating endothelial progenitor cells from the blood of patients with classic KS (cKS) and assessed their status of KSHV infection.

## Methods

### Study subjects

Sixteen patients with cKS were included in the study, 10 males and 6 females, mean age 70 years (range 47–82). All patients had histologically confirmed diagnosis of KS, were positive for anti-HHV8 antibodies and negative for HIV. Patients in systemic chemotherapy were excluded. Staging was performed in accordance with our classification that takes into account the prevalent type of lesions, localization, clinical behaviour, evolutive pattern and presence of complications [7], [8]. Late endothelial progenitor cell (late-EPC) cultures were performed at a single time point on fresh peripheral blood samples from all the patients; staging at this time is reported in Table 1. Seven of these patients underwent KSHV-DNA analysis and titration of KSHV-specific antibodies; one healthy KSHV-seronegative control was included for these analyses. Ethics approval was obtained from the local Institutional Review Committee and a signed informed consent was obtained from all participants.

**Table 1:** Clinical characteristics of patients with cKS at the time of late-EPC culture.

Characteristics	Overall patients
No. of patients	16
Age, yr^a^	70.0±2.4
Sex, no.	
Male	10
Female	6
KS stage^b^, no.	
I: maculo-nodular	
A	4
B	5
II: infiltrative	
A	2
B	1
III: florid	
A	1
B	2
IV: disseminated	
A	0
B	1

a Mean±standard error.

b cKS patients were classified according to our classification that takes into account the prevalent type of lesions, localization, clinical behaviour, evolutive pattern and presence of complications [7], [8].

A = slow evolution; B = rapid evolution; rapid denotes an increase in the total number of nodules/plaques or in the total area of plaques in the three months following the last examination.

### Cultures of late-endothelial progenitor cells

Late-EPCs were generated from peripheral blood mononuclear cells (PBMCs) of cKS patients according to methods described by Ingram [9], with minor modifications. Briefly, PBMCs were obtained by Ficoll density gradient centrifugation (Cedarlane, Hornby, Canada) [10] from 30 ml of fresh venous peripheral blood, resuspended in EGM-2 medium (Cambrex Bio Science, Walkersville, MD) and seeded onto six-well tissue culture plates (5×10^6^ cells/well) precoated with human fibronectin (1 µg/cm^2^, Sigma-Aldrich, St. Louis, MO). Four to eight wells (mean 6.07±s.e. 0.37) were plated for each patient. In two experiments, PBMCs underwent separation of CD14+ and CD14- fractions using immunomagnetic selection with mini-magnetic-activated cell sorter (MACS) cell isolation kit (Miltenyi Biotec, Bergisch Gladbach, Germany) according to the manufacturer's instructions. After 3 days of culture, nonadherent cells and debris were aspirated, adherent cells were washed with EGM-2 medium, and EGM-2 medium was added to each well. Medium was changed on day 7 and every 3 days until the first passage. Late-EPC colonies were identified by visual inspection using an inverted microscope. Late-EPCs were released from the original tissue culture plates by trypsinization (trypsin 0.25%) (Euroclone, Wetherby, UK), resuspended in EGM-2 medium, and plated onto 25 cm^2^ tissue culture flasks precoated with fibronectin for further passages. Multiple colonies from the same patient were pooled at this step. In one single experiment, late-EPCs obtained from a KS patient underwent cell sorting of CD146+ cells (EPICS ALTRA Cell Sorter; Beckman-Coulter, Fullerton, CA). Purity of CD146+ cells before and after sorting was 98.7% and 100%, respectively. This highly pure population of CD146+ late-EPCs was replated and maintained in culture for further two weeks for virus analyses.

### Characterization of late-endothelial progenitor cells

Cells were cultured onto fibronectin-coated, 2-chamber Lab-Tek slides (Nalge NUNC International, Rochester, NY) and attached cells were incubated with 10 µg/ml Dil-acetylated-low-density lipoprotein (Dil-ac-LDL) (Molecular Probes, Eugene, OR) in EGM-2 medium for 1 hour at 37°C. Cells were then fixed with 2% formaldehyde for 10 minutes and incubated for 1 hour with fluorescein-isothiocyanate (FITC)-labeled Ulex Europaeus Agglutinin-1 (UEA-1; Sigma-Aldrich). Alternatively, cells were fixed with methanol, permeabilized with 0.1% Triton X-100, blocked with 10% normal goat serum (Dako, Hamburg, Germany) and incubated with purified antibodies against human von Willebrand Factor (vWF), endothelial nitric oxide synthase (e-NOS) or caveolin-1 (Cav-1) (Becton-Dickinson, San Jose, CA), followed by appropriate FITC- or phycoerythrin (PE)-conjugated secondary antibodies (Jackson Immunoresearch, West Grove, PA). Slides were mounted in anti-fading mounting media containing DAPI (Vectashield; Vector Laboratories, Burlingame, CA) and examined by conventional fluorescence-microscopy (Leitz Dialux 22 microscope system).

For antigen detection by flow cytometry, 5×10^5^ late-EPCs were incubated with different anti-human monoclonal antibodies (mAbs) for 30 minutes at 4°C in the dark. The following FITC-, PE-, allophycocyanin (APC)- or biotin-conjugated mAbs were used: anti-CD31, -CD45, (Becton-Dickinson); -kinase insert domain receptor (KDR) (Sigma-Aldrich), -CD105 (Euroclone), -CD14 (Caltag Laboratories, Burlingame, CA), -CD146 (Chemicon, Temecula, CA), -CD34 (Miltenyi Biotec). The FASER technique was performed for CD34 detection according to manufacturer's instructions (Faser kit-APC, Miltenyi Biotec). Isotype-matched irrelevant mAbs were used as negative controls. Data were acquired on a FACSCanto flow cytometer (Becton-Dickinson) and analyzed using FACSDiva software. Cells were electronically gated according to light scatter properties to exclude cell debris.

Matrigel assays were performed as described [9]. Late-EPCs were seeded onto 96-well tissue culture plates coated with 70 µl Matrigel (Becton-Dickinson) at a cell density of 2×10^4^ cells per well, and incubated at 37°C. Cells were observed during the following 12–72 hours with inverted microscopy for capillary-like formation.

### Assessment of KSHV infection

Plasma samples were analyzed for the presence and titers of antibodies to a latency-associated nuclear antigen (LANA) and to a lytic phase-associated structural protein encoded by ORF65, as previously described [11]. Antibody titers were calculated as the reciprocal of the highest plasma dilution giving positive results. PBMC and late-EPC cultures from 7 cKS patients and from a healthy control were tested for the presence and load of KSHV DNA sequences. DNA was extracted from at least 2×10^5^ cells and from 200 ul of DNAse-treated culture supernatants as previously described [12]. KSHV viral load was measured by quantitative real-time polymerase chain reaction (PCR), as reported elsewhere [12]. To induce KSHV lytic replication, late-EPCs were incubated for 48 hours either with 3 mM *n*-butyrate (Sigma-Aldrich), or with 20 ng/ml of phorbol 12-myristate 13-acetate (TPA, Sigma-Aldrich) [12]. Alternatively, late-EPCs were exposed to hypoxia by incubation for 96 hours in anaerobic conditions with less than 2% oxygen (GasPack-Pouch Systems, Becton-Dickinson) [13].

### Statistical analysis

The Wilcoxon signed-rank test was used to analyze the statistical significance of data obtained in the *in vitro* induction of KSHV lytic replication. Analysis was performed with OPENSTAT3 software.

## Results

### Late-endothelial progenitor cell cultures from classic Kaposi's sarcoma patients

**Figure 1:**
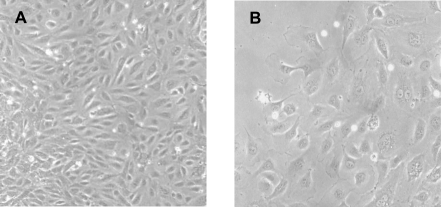
Late-EPCs can be cultured from peripheral blood mononuclear cells of patients with cKS. A representative phase-contrast photograph of a late-EPC colony, identified as a well-circumscribed monolayer of cobblestone-appearing cells, is shown. Similar colonies were obtained from 15 different patients. A) ×100 magnification, B) ×200 magnification. Late-EPCs were photographed using a Leitz Diavert microscope system.

**Figure 2:**
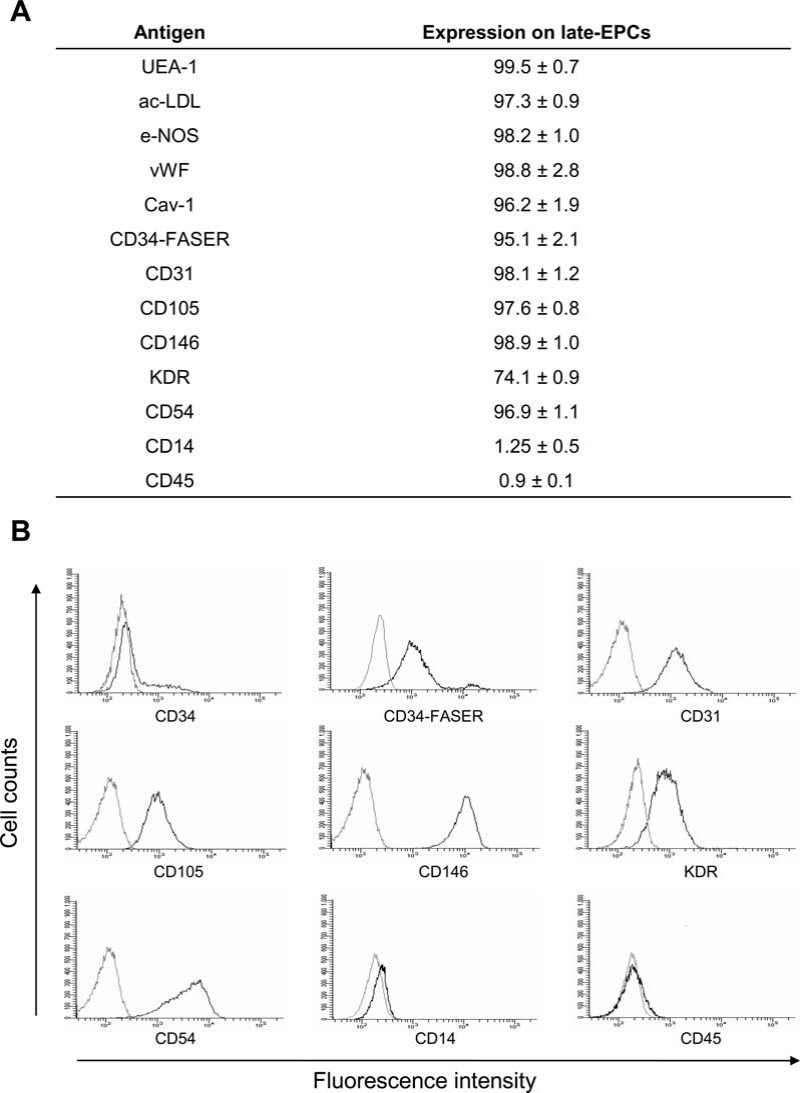
Immunophenotypic characterization of late-EPCs obtained from 15 patients with cKS. Late-EPCs express high levels of endothelial antigens but lack leukocyte markers. A) Percentage of positive cells for the indicated antigens, data expressed as mean±standard error. B) Representative flow cytometry analysis. Note that binding of UEA-1, uptake of ac-LDL and staining of e-NOS, vWF and Cav-1 were examined by conventional fluorescence-microscopy. UEA-1 = Ulex Europaeus Agglutinin-1; ac-LDL = acetylated-low-density lipoprotein; e-NOS = endothelial nitric oxide synthase; vWF = von Willebrand Factor; Cav-1 = caveolin-1.

**Figure 3:**
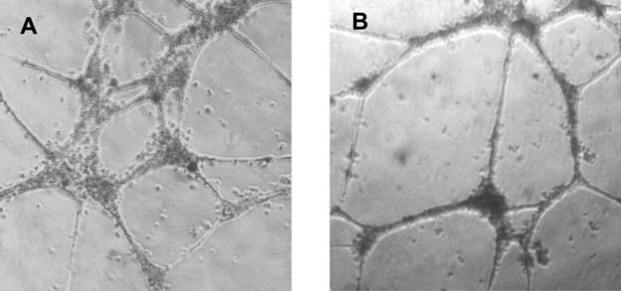
Late-EPCs obtained from patients with cKS form capillary-like structures in vitro. Late-EPCs cultured in Matrigel gave rise within 12 hours of incubation to vascular structures that were quite similar to those formed by control endothelial cell lines. A) A representative phase-contrast photograph of the capillary-like structures formed by late-EPCs from a patient with cKS (×40 magnification) is shown. B) For comparison, a photograph of the structures formed by ECV 304 cell line (×40 magnification) is shown. Capillary-like structures were photographed using a Leitz Diavert microscope system.

Late-EPCs were obtained from the PBMC of 16 cKS patients whose characteristics are reported in Table 1. Small colonies originated from adherent cells and were apparent after 11–26 days of culture; the mean number of colonies generated from 10^6^ PBMCs was 0.090±0.020, equivalent to 2.81±0.39 colonies from each patient. Late-EPCs proliferated slowly during the first weeks, but then proliferated more rapidly and could be serially expanded in vitro through multiple passages, up to 7–10 weeks, when they finally stopped growing and entered senescence. During these passages the cells retained their endothelial morphology, forming cobblestone-like monolayers (Fig. 1). Late-EPCs stained with UEA-1, efficiently took up ac-LDL and expressed high levels of the endothelial antigens e-NOS, vWF, Cav-1, CD31, CD105, CD146, KDR and CD54, but were negative for the leukocyte markers CD45 and CD14 (Fig. 2A). According to previous observations [14], the expression of CD34 on late-EPC surface was at low intensity and could therefore be better detected when the highly sensitive FASER technique, instead of conventional cytometry, was used (Fig. 2B). Finally, within 12 hours of incubation on Matrigel, late-EPCs formed characteristic tube-like structures assembled in a branching reticular network, indistinguishable from those generated by a control endothelial cell line (Fig. 3). On the whole, these data indicate that the cultures obtained from patients with cKS satisfied all requisites to be defined late-EPCs.

Because it has been demonstrated that late-EPCs develop exclusively from the CD14- fraction of PBMCs [15], the CD14+ and CD14- fractions were separated from PBMCs of two cKS patients on day 0 (>98% purity by flow cytometry) and were cultured independently. As expected, late-EPC colonies quite similar to those obtained from unfractionated PBMCs developed in CD14- but not in CD14+ cultures (not shown).

### Analysis of KSHV presence and viral load quantification

**Figure 4:**
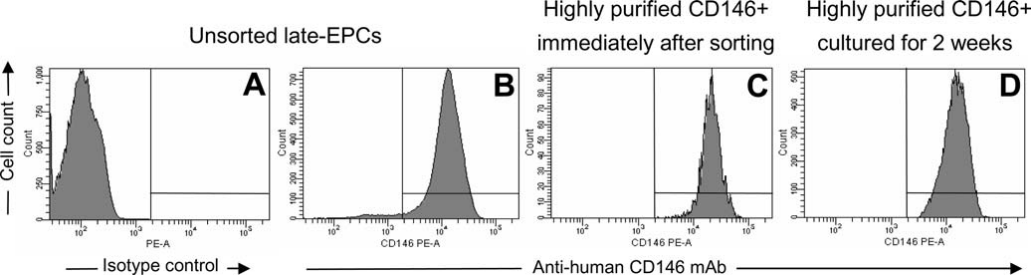
Flow cytometry analysis showing CD146 expression on different late-EPC populations. To confirm that late-EPCs with proven endothelial phenotype could support KSHV infection, late-EPCs from one cKS patient underwent cell sorting of CD146+ cells and were cultured for further 2 weeks. Analysis of unsorted late-EPCs stained with isotype control (A) or anti-human CD146 mAb (B); analysis of highly purified CD146+ late-EPCs stained with anti-human CD146 mAb immediately after sorting (C) or after 2 weeks of culture (D).

KSHV DNA presence and load was determined by real-time PCR in PBMCs and late-EPC cultures from 7 cKS patients, and in one KSHV-seronegative healthy control, as shown in Table 2. PBMC samples from all cKS patients were found to harbor KSHV DNA sequences [7–359 KSHV genome equivalents (GE)/10^5^ cells]. Real-time PCR analyses performed on late-EPC cultures revealed the presence of detectable amounts of KSHV genomes in all cultures obtained from the cKS patients, whereas late-EPCs from the healthy control were found to be negative. Moreover, KSHV DNA was detected at variable levels in DNA extracted from culture supernatants, suggesting that a quote of KSHV lytic infection may spontaneously occur in these cells. To further confirm these data and possibly analyze the trend of KSHV infection in these cells, late-EPCs obtained from two patients were analyzed in multiple passages. As shown in Table 2, late-EPCs from patient KS 4 showed a peak replication level (1332 GE/ml) in culture supernatant at day 34, followed by high levels of viral genomes in cellular DNA obtained in the following passage, suggesting the propagation of viral progeny to uninfected cells. Moreover, in another patient (KS 6) KSHV DNA could be detected in relatively high amounts even in cells and supernatants collected from late-EPC cultures after longer periods of time (day 71), indicating that late-EPCs may retain KSHV infection until senescence. Because none of the endothelial markers used to characterize our cultures was singularly expressed on the totality of late-EPCs (each single marker being expressed by up to 98.9±1% of cells, Fig. 2A), we wondered whether cells expressing endothelial markers could be responsible for KSHV infection in our cultures. Therefore, in one experiment late-EPCs (from patient KS 7) were sorted for the endothelial marker CD146 [15], [16], replated and analysed for viral load and release before and after cell sorting, strictly maintaining the same culture conditions. Culture of sorted CD146+ late-EPCs gave rise to cobblestone-like monolayers of cells morphologically identical to cultures of unsorted cells. The persistence of CD146 positivity of sorted CD146+ late-EPCs was confirmed after 2 weeks of culture, before performing viral analysis (Fig. 4). As shown in Table 2, the levels of viral genomes in cellular DNA remained similar before and after sorting in basal conditions (250 and 271 KSHV GE/10^5^ cells, respectively). Also the amounts of viral genomes spontaneously released by late-EPCs in the supernatants were similar before and after sorting (584 and 667 KSHV GE/ml, respectively), thus suggesting that the negligible proportion of CD146-negative cells did not represent the source of virus in our cultures.

**Table 2:** KSHV-DNA in peripheral blood mononuclear cells and in late-EPC cultures from patients with cKS according to their clinical stage and KSHV serology.

						PBMCs	late-EPCs	late-EPC supernatants	
				KSHV serology^a^		KSHV-DNA	KSHV-DNA	KSHV-DNA	
Patient	Gender	Age	KS stage^b^	Anti-ORF65	Anti-LANA	GE/10^5^ cells	GE/10^5^ cells	GE/ml	Day of culture
KS 1	F	76	1A	400	1600	17	18	335	23
KS 2	F	72	1B	100	100	51	145	468	30
KS 3	F	62	1A	800	12800	106	74	760	31
KS 4	M	80	3B	–	25600	81	104	1332	34
							1327	959	42^c^
							64	594	48^c^
KS 5	M	72	1B	–	6400	7	109	564	43
KS 6	M	62	2B	6400	400	359	182	906	47
							335	1329	71^c^
KS 7	M	77	1B	–	3200	187	250	584	21
							271^d^	667^d^	35
Healthy control	F	68		–	–	0	0	0	38

a Antibody titers were calculated as the reciprocal of the highest plasma dilution giving positive results.

b cKS patients were classified according to our classification that takes into account the prevalent type of lesions, localization, clinical behaviour, evolutive pattern and presence of complications [7], [8].

c In two patients the presence of KSHV-DNA was determined in multiple passages of unstimulated late-EPC cultures.

d KSHV-DNA determined in highly pure CD146+ late-EPCs, maintained in culture for further 2 weeks after CD146 sorting.

KSHV = Kaposi's sarcoma-associated herpesvirus; PBMCs = peripheral blood mononuclear cells; late-EPC = late-endothelial progenitor cell;

cKS = classic Kaposi's sarcoma; LANA = latency-associated nuclear antigen; GE = genome equivalents.

### Induction of KSHV lytic cycle

**Figure 5:**
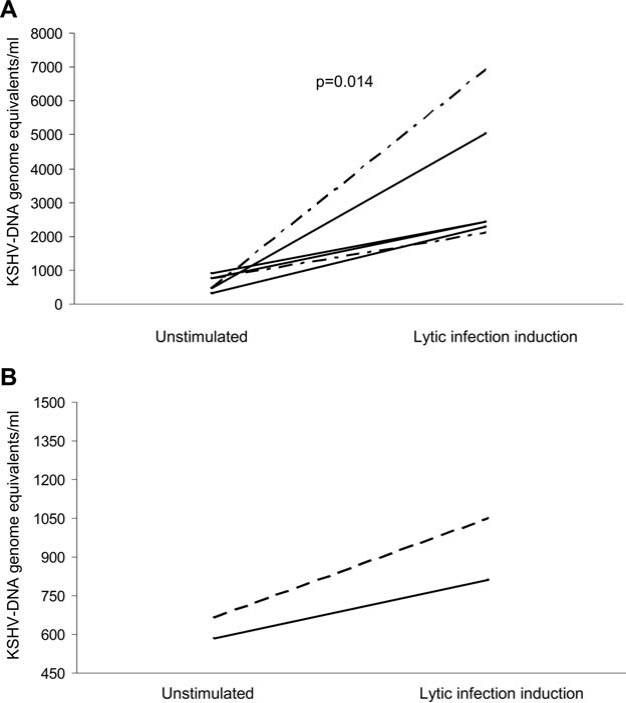
Late-EPCs obtained from patients with cKS support KSHV productive replication. A) To induce KSHV lytic replication, late-EPCs from cKS patients underwent treatment with *n*-butyrate (5 patients, solid lines) or TPA (2 patients, hatched lines) for 48 hours. Multiple colonies from each patient were pooled before treatment. B) To confirm that late-EPCs with proven endothelial phenotype could support KSHV lytic replication, late-EPCs from one cKS patient underwent cell sorting of CD146+ cells. Unsorted late-EPCs (solid line) or highly purified CD146+ late-EPCs from the same cKS patient were cultured for further 2 weeks after sorting (hatched line) and underwent treatment with *n*-butyrate for 48 hours. In any case, KSHV genomes were analyzed by real-time PCR in DNA extracted from late-EPC supernatants. *P* value was determined using the Wilcoxon signed-rank test.KSHV genomes were analyzed by real-time PCR in DNA extracted from culture supernatants. KSHV = Kaposi's sarcoma-associated herpesvirus; TPA = phorbol 12-myristate 13-acetate.

To induce the viral lytic cycle in KSHV-infected late-EPCs, cultures from two patients were treated in parallel with *n*-butyrate or TPA, whereas cultures from three other patients had sufficient cells to be treated only with *n*-butyrate. KSHV genomes were measured by real-time PCR in culture supernatants 48 hours after induction. As reported in Fig. 5A, induction of KSHV lytic program by chemical treatment lead to a statistically significant increase in virus release in culture supernatants (Wilcoxon signed-rank test, *P* = 0.009), suggesting that late-EPCs may support KSHV productive replication. As shown in Fig. 5B, *n*-butyrate treatment of sorted CD146+ late-EPCs (from patient KS 7) increased the release of KSHV genomes in the supernatants to the same extent as unsorted cells (1050 and 812 KSHV GE/ml, respectively), thus suggesting that late-EPCs with proven endothelial phenotype can support viral lytic induction.

The effects of late-EPC exposure to prolonged hypoxia, which may act in vivo as a cofactor in the development of KS lesions, were evaluated in 2 patients. In both cultures, hypoxia induced an increase in KSHV levels in culture supernatants. In facts, late-EPCs from patient KS 5 showed a 5-fold increase in virus release (from 906 to 4454 GE/ml), whereas a moderate increment was observed in the culture of patient KS 6 (from 564 to 690 GE/ml). Late-EPCs obtained from the healthy negative control were found to be repeatedly negative for KSHV DNA sequences also in hypoxic culture conditions (not shown).

## Discussion

In this report, we provide evidence that late-EPCs from the peripheral blood of patients with cKS harbor KSHV, may support lytic replication and may act, therefore, as viral reservoirs. The novelty of this finding resides in the nature of late-EPCs. Among other cell populations with endothelial features circulating in adult peripheral blood, late-EPCs, also called outgrowth endothelial cells (OECs) [14] or endothelial colony-forming cells (ECFCs) [9], are the cells that contribute more directly to neovascularization [17]. Late-EPCs derive from the CD14- fraction of circulating mononuclear cells, may be enriched in cells that express some combination of CD34 and KDR, uniformly express endothelial but not leukocyte markers and are able to form capillary tubes in vitro [18]–[20]. They form colonies of endothelial cells endowed with a late and robust proliferative potential, so that they can be passaged over 2 months ex vivo. Because they act in many aspects like classical primitive hematopoietic progenitor cells, late-EPCs might represent a major source of endothelial progenitors in vivo [19], [21], [22], and may therefore be an optimal model for the study of KS. Accordingly, it has been recently demonstrated that murine bone marrow-derived endothelial progenitors, analogous to human late-EPCs, transfected in vitro with a KSHV artificial chromosome induce KS-like tumors in mice [23].

First of all, in this study we demonstrate that late-EPCs can be cultured from the peripheral blood of patients with cKS. Moreover, our results clearly indicate that late-EPCs from cKS are infected by KSHV and may retain the virus in long-term cultures, as late-EPCs from the totality of cKS patients studied resulted to harbor variable amounts of viral genomes in cultures tested up to over 2 months.

Infected spindle cells explanted from KS biopsies are known to rapidly segregate and loose KSHV genomes when placed in culture [24]–[26]. It is possible that the ability of late-EPCs to retain longer KSHV episomes may be related to the differentiative stage of these cells, that are not mature endothelial cells, but progenitors endowed with high replicative potential. Another population of circulating endothelial-featured cells able to support viral replication had been reported by Monini and colleagues [2]. They derived spindle-like endothelial macrophages from PBMCs of KS patients able to support KSHV infection up to 4 weeks. Although these cells differed from late-EPCs as they expressed CD45 and CD14 and were considered of monocytic origin, this pioneeristic study greatly contributed to the idea that circulating endothelial precursors may act as reservoir for KSHV infection.

The detection of viral progeny released in all the supernatants of unstimulated cultures indicates that late-EPCs may support the lytic replication program of KSHV and this finding may alternatively suggest that the virus is propagated in culture by cycles of de novo infection of late-EPCs. Accordingly, when KSHV DNA was analyzed in subsequent passages of unstimulated late-EPCs from patient KS 4, we could observe that the levels of viral genomes may change over time. Whether these fluctuations may reflect cycles of productive viral replication that may be responsible for the continuous infection of endothelial progenitors may deserve further investigation.

Moreover, our results indicate that the basal level of lytic replication can be further increased not only by chemical treatment but also by hypoxic conditions. This finding is worthy relevant to the role that lytic KSHV replication is supposed to play in the promotion of KS tumorigenesis by sustaining the population of latently infected cells [23], [27]. And it is still more relevant because hypoxia-induced KSHV reactivation, demonstrated in vitro in PEL cell lines [28], has been suggested to contribute to the tendency of KS to occur on the lower legs due to poor circulation, particularly in cKS patients [29]. In this respect, hypoxia may be considered as a KS cofactor, and the ability of late-EPCs to support viral lytic reactivation in hypoxic conditions may have particular relevance in vivo.

In addition, the fact that late-EPCs harbor moderate amounts of viral genomes in unstimulated conditions may be partly related to the type of KS patients selected for the study. In fact, to avoid the interference of confounding factors related to iatrogenic or AIDS-related KS, we studied patients affected by the classic, Mediterranean variant of the disease that is typically characterized by a poorly aggressive course and a relatively low viral load [16], [30], as confirmed in the PBMCs of our patients. The detection of moderate amounts of viral genomes in our cultures also suggests that only a small proportion of late-EPCs may be infected with KSHV. Alternatively, it may be hypothesized that the source of virus in our cultures may be represented by the negligible percentage of cells lacking endothelial markers. The demonstration that KSHV genomes could similarly be detected in unsorted or CD146+ highly purified late-EPCs does not definitely exclude this possibility but renders it unlikely.

Endothelial progenitors are thought to be contained, together with hematopoietic stem/progenitor populations, within the pool of circulating CD34+ cells [31]. It has been reported that circulating CD34+ cells, that we recently demonstrated to be increased in patients with cKS [8], [32], may harbor KSHV [33]. Similarly, it has been demonstrated that circulating CD146+ cells, which contain endothelial progenitors but also mature endothelial cells [34], may harbor the virus, too, in KS patients [16]. However, cell subpopulations of CD34+ or CD146+ preparations were not further characterized and therefore both the observations are compatible with, but not demonstrative for, KSHV infection of endothelial progenitors.

In conclusion, in this study we suggest that circulating endothelial progenitor cells cultured from the peripheral blood of cKS patients as late-EPCs are infected by KSHV and can support viral lytic replication. This finding may be of crucial importance for the comprehension of KS pathogenesis, because late-EPCs may likely represent the KSHV-infected circulating precursors of KS spindle cells that, as recently suggested by Gill, may home to the permissive site and propagate to produce KS lesions [35]. A definitive demonstration of this hypothesis will need experimental models of in vivo tumorigenesis.
